# An Unusual Presentation of Duodenal Metastasis of a Previously Undiagnosed Primary Lung Adenocarcinoma

**DOI:** 10.7759/cureus.24958

**Published:** 2022-05-13

**Authors:** Sai Karthik Kommineni, Syed M Sadiq, Tharun Bandarupalli, Deidre M Pierce

**Affiliations:** 1 Internal Medicine, East Tennessee State University Quillen College of Medicine, Johnson City, USA

**Keywords:** lung cancer, metastatic adenocarcinoma of lung, metastatic non-small cell lung cancer, abdominal abscess, adenocarcinoma lung

## Abstract

A cancer diagnosis can be a frightening experience for both patients and their families. It is at this juncture that a prompt diagnosis of cancer becomes vital. Lung cancer is the leading cause of cancer-related deaths in men and women due to its aggressive nature and widespread metastasis. A metastatic lesion leading to the diagnosis of cancer followed by identifying the primary source is not unusual. Still, it is very uncommon for cancer to present with signs and symptoms of an infectious abscess. We report a case of metastatic adenocarcinoma of the lung that presented as an abdominal abscess. This article aims to bring more awareness among the medical community to include cancer in the differential for early diagnosis of potentially lethal cancer.

## Introduction

Lung cancer is the leading cause of cancer-related deaths, accounting for 23% of all cancer-related deaths [1]. Lung cancer comprises two significant subtypes: non-small cell lung cancer (NSCLC) and small cell lung cancer (SCLC). NSCLC encompasses adenocarcinoma, squamous cell carcinoma, and large cell carcinoma. NSCLC is the most common type of lung cancer and accounts for 84% of all lung cancer diagnoses [2]. The most common extrapulmonary metastases from adenocarcinoma sites are lymph nodes, liver, brain, bones, and adrenal glands. The solitary metastasis to the small bowel is rare and accounts for 0.5-2% of cases [3]. We describe an atypical presentation of metastatic lung adenocarcinoma.

## Case presentation

**Figure 1:**
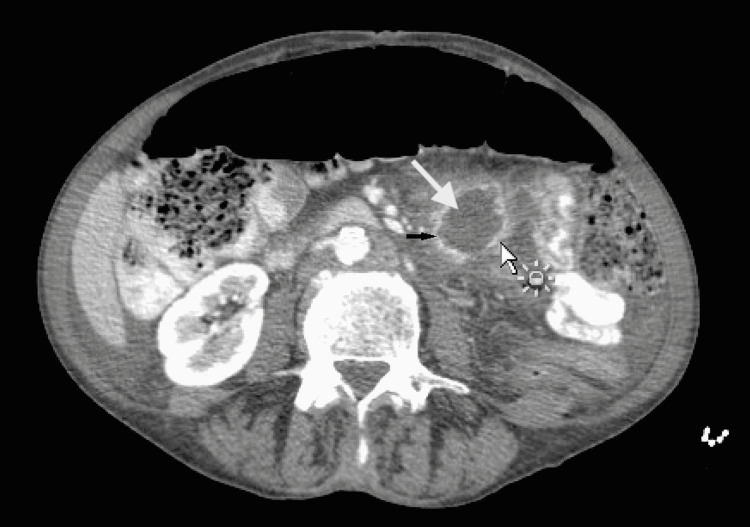
CT of the abdomen and pelvis with contrast showing 3.2 x 3.8 x 2.7 cm irregularly marginated peripherally enhancing fluid collection in the left mid abdominal mesentery with prominent surrounding inflammation consistent with abscess. A white short arrow shows surrounding inflammation, a white large arrow shows necrotic material, and a black arrow shows ring enhancement.

A 61-year-old female with a medical history significant for tobacco use for several years presented with diffuse lower abdominal pain, fever, chills, and loss of appetite. At the time of presentation, her vital signs were stable with a blood pressure of 110/63, heart rate of 98, temperature of 98.7, and saturation of 99% on room air. Physical exam was significant for left lower quadrant tenderness but otherwise unremarkable. A CT scan of the abdomen was performed, which revealed a 3 cm (about 1.18 in) fluid-filled ring-enhancing lesion concerning abscess (Figure 1).

**Figure 2:**
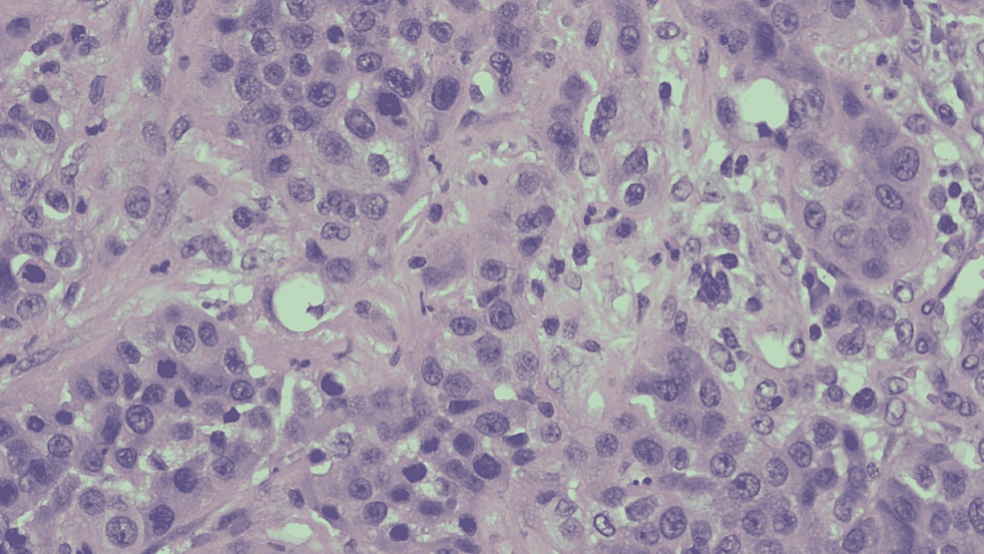
Hematoxylin and eosin (H&E) stain of the biopsied mass at high power magnification.

The patient was started on broad-spectrum antibiotics, and surgery was consulted for a diagnostic laparoscopy with incision and drainage. A firm lesion of the proximal small bowel of about 5 cm (about 1.97 in) was found during laparoscopy. The surgery was converted to exploratory laparotomy. On inspection during laparotomy, we found a firm mass of the small bowel just distal to the ligament of Treitz with extensive involvement of mesentery. Given the extensive involvement, resection of the mass could not be performed, so a biopsy was done and was sent for histology. The histological sections of the biopsied lesion showed poorly differentiated adenocarcinoma mixed with abundant necrotic cells (Figure 2).

**Figure 3:**
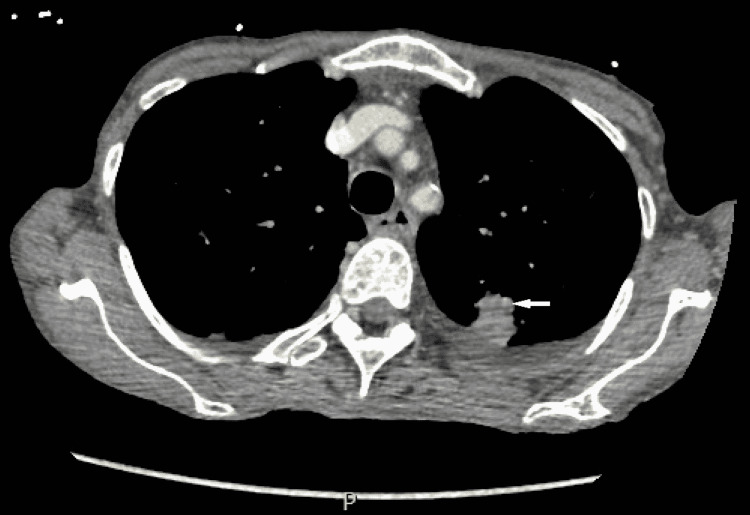
CT scan of the chest showing 21 mm (about 0.83 in) subpleural density in the posterior aspect of the left upper lobe. A white arrow shows subpleural density.

Extensive immunohistochemistry was performed, and the malignant cells were positive for cytokeratin 7 (CK7), MOC-31, and thyroid transcription factor 1 (TTF-1). Given the strong TTF-1 positivity, the findings were most consistent with metastatic adenocarcinoma of the lung. A CT scan of her chest was done following the biopsy findings, and it showed 21 mm (about 0.83 in) subpleural density in the posterior aspect of the left upper lobe (Figure 3). A metastatic lung adenocarcinoma was diagnosed, and the results were explained to the patient. The patient was discharged from the hospital with medical and surgical oncology follow-up.

## Discussion

In the United States, lung and bronchial cancer incidence and mortality rates have declined from 1999 to 2018 [4]. However, it is still the most common cause of cancer-related deaths. The overall five-year survival rate is less than 15%, even worse with 7% in those who have metastases [4]. When diagnosed, lung adenocarcinoma is usually metastasized via hematogenous or lymphatic routes to the brain, bone, or adrenal glands [5].

Most metastatic lesions from primary lung cancer to the gastrointestinal (GI) tract are found during the autopsy. Although a rare presentation, non-small cell lung carcinoma is most likely to spread to either gastric or colonic mucosa [5]. A large metanalytic study within the GI system showed that more than half of lung cancer metastases are in the small intestine, with the least common site involving the duodenum [6]. Metastasized lung carcinoma to the small bowel in symptomatic patients usually presents with abdominal pain, perforation, GI bleeding, and intussusception/obstruction, which warrants emergent surgical intervention [7].

**Figure 4:**
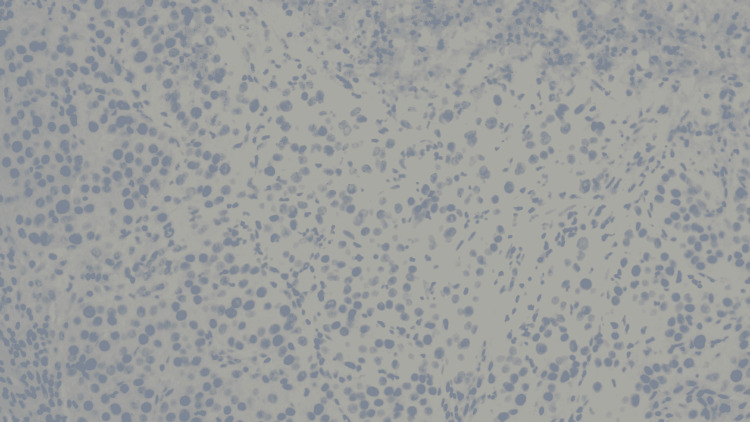
CDX2 testing showing negative CDX2, which is a marker used to determine the mass of gastrointestinal origin.

**Figure 5:**
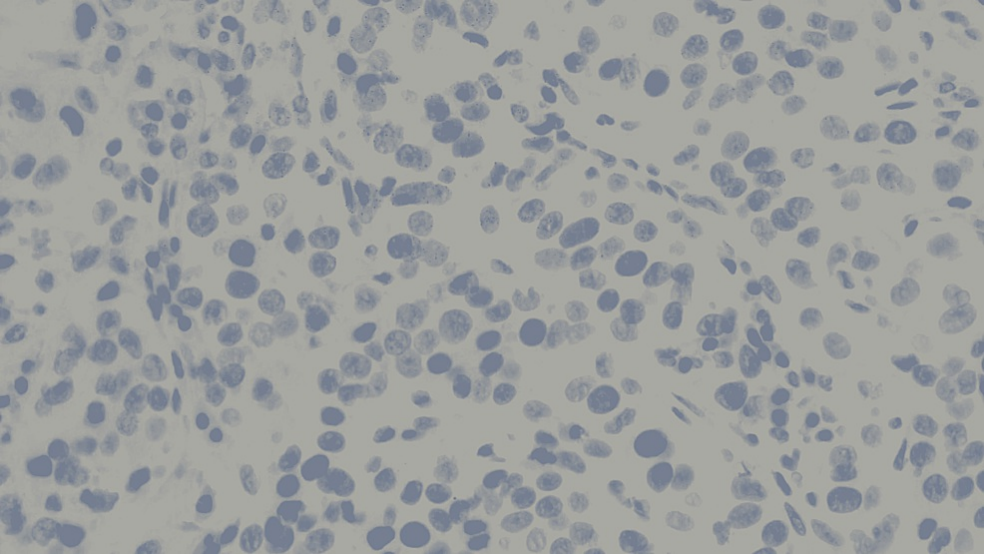
DOG1 testing showing negative DOG1, which is a gastrointestinal stromal tumor (GIST) marker that was ordered because of the location to exclude an epithelioid GIST.

Risk factors for primary lung adenocarcinoma include tobacco abuse, radon exposure, environmental exposures, and family history. Our patient did have an extensive smoking history but no respiratory symptoms. She had not undergone any lung cancer screening, so there was a slight suspicion of any lung pathology. Lung carcinoma is an insidious disease that, when diagnosed, is most common in stage IV [5]. Sensitive immunomarkers such as TTF-1 and CK7 will help us differentiate the neoplasm from primary lung adenocarcinoma. CK7 is primarily positive in many adenocarcinomas. In our patient presenting with GI symptoms, we tested for CDX2 and DOG1. CDX2 is a tumor marker for primary GI origin (Figure 4), and DOG1 is a marker for GI stromal tumors (Figure 5), which were both negative in our patient, and it helped us rule out primary GI neoplasm.

**Figure 6:**
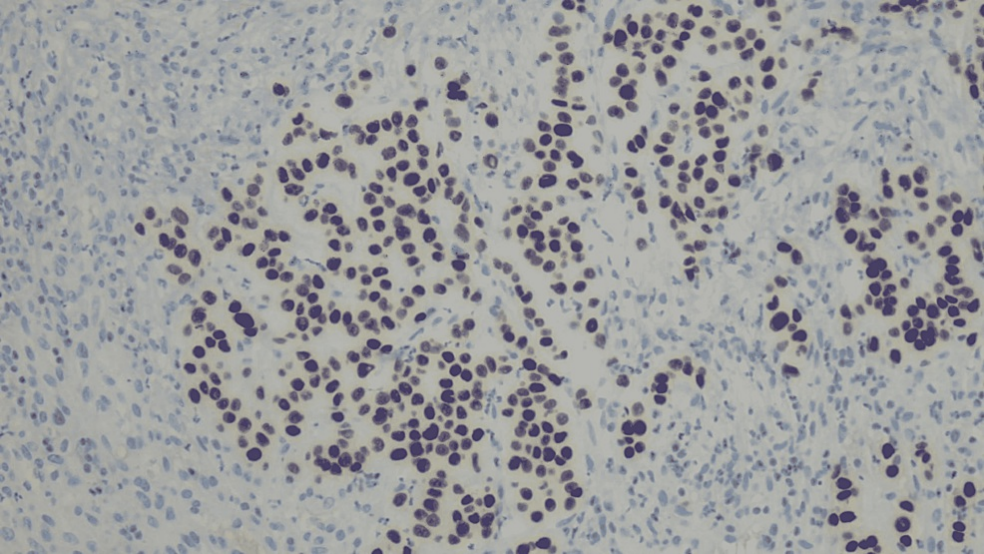
TTF-1 testing showing positive TTF-1, which is positive in lung and thyroid cancers. TTF-1: thyroid transcription factor 1.

**Figure 7:**
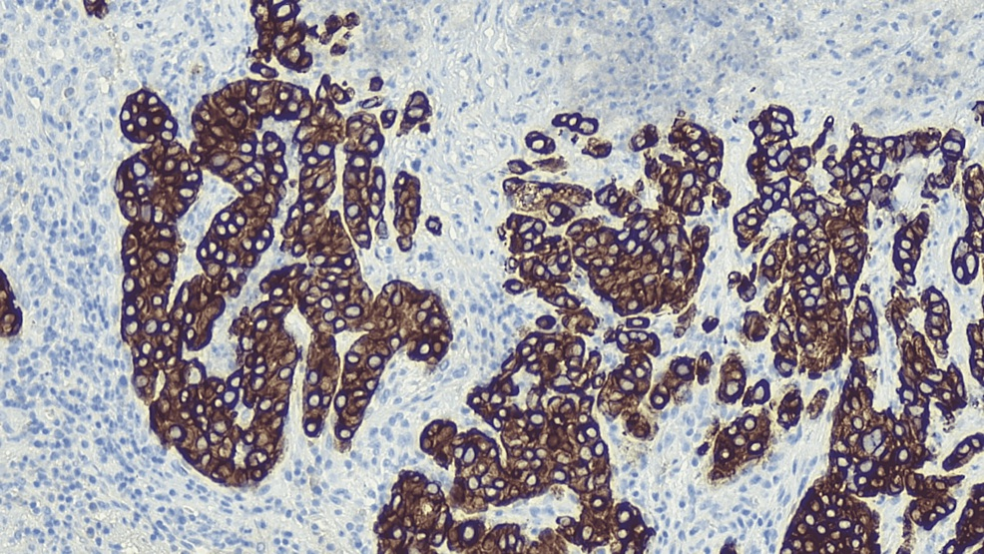
CK7 testing showing positive CK7, which is typically positive in adenocarcinomas of various sites including lungs. CK7: cytokeratin 7.

**Figure 8:**
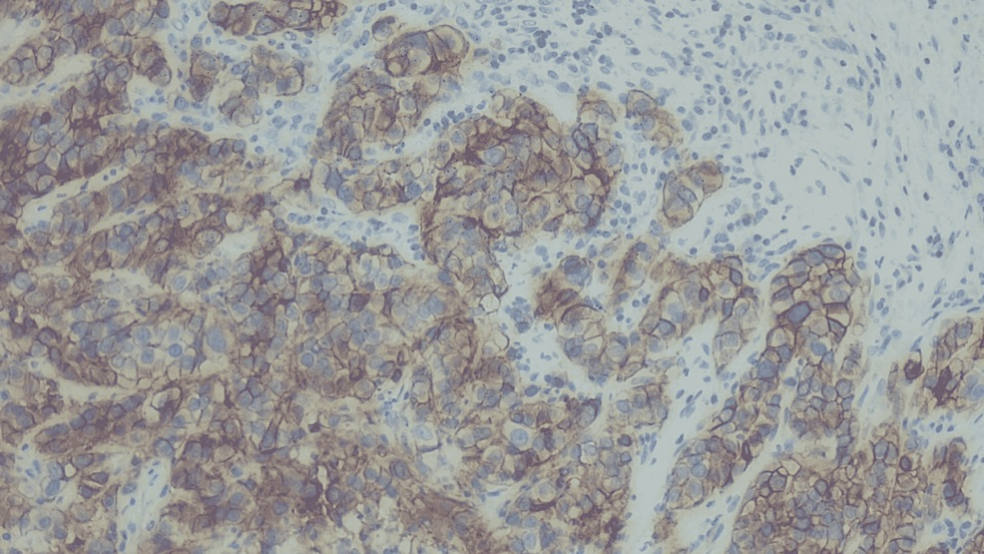
MOC-31 testing showing positive MOC-31, which is used when the differential includes adenocarcinoma and mesothelial carcinoma. Positive in adenocarcinoma of the lung.

We then tested for markers TTF-1 (Figure 6) and CK7 (Figure 7), which allowed us to determine the lung as the primary source and differentiate it from thyroid neoplasms [8]. Once lung origin was determined, tumor marker MOC-31 (Figure 8) helped differentiate adenocarcinoma from mesothelioma of the lung [9].

Treatment of lung adenocarcinoma depends on the stage of the disease. The American Joint Committee on Cancer (AJCC) staging is used to determine the severity of the disease. Tumors in stages I, II, and IIIA are considered limited invasive tumors or limited nodal diseases. The treatment of a tumor in these stages depends on resectability. If operable, surgical treatment is recommended with lymph node sampling. In patients who are not surgical candidates, definitive radiotherapy with adjuvant chemotherapy is recommended based on the patient’s tumor characteristics [10,11]. Stages IIIB and IV are considered non-resectable and are treated with chemoradiation. The tumors are tested for epidermal growth factor receptor (EGFR) sensitizing mutations and anaplastic lymphoma kinase (ALK) mutation. Patients positive for EGFR are treated with tyrosine kinase inhibitors, and those with ALK mutation are treated with ALK inhibitors as first-line agents [4].

The survival rate of metastatic lung adenocarcinoma is less than any other stage in the literature. Specifically, with metastatic lesions to the abdomen, the survival rate is less than any other organ. GI metastases usually have extensive involvement in the submucosa and surrounding lymph nodes, and the median survival is between one and 15 months with GI metastases. The GI tract is the only organ labeled as a poor prognostic factor for the site of metastasis [12]. Although radiation and chemotherapy should be considered case-by-case, surgical resection is often a palliative procedure due to poor prognostic factors.

## Conclusions

There are no reported cases of primary lung carcinoma presenting as abdominal abscess so far in our review. In our case, the patient was presented with persistent leukocytosis without significant anemia. A CT of the abdomen and pelvis showed a 3 cm (about 1.18 in) ring-enhancing fluid collection within the small bowel, supporting the diagnosis of an abscess.

Adenocarcinoma of the lung is a slowly progressive disease with a subtle presentation. As in our case, an uncommon site metastasis to the abdomen, initially presenting as an abscess, led to a neoplasm diagnosis. This unusual presentation should encourage clinicians to include a broad differential, including neoplastic origin.
